# Molecular Mechanisms Underlying Substance Transport, Signal Transduction, and Anti-Stress Regulation, as Well as Anti-Alkaline Regulation via *Bursicon* in the Cerebral Ganglion of Chinese Mitten Crab *Eriocheir sinensis* Under Alkaline Stress

**DOI:** 10.3390/biology14010084

**Published:** 2025-01-16

**Authors:** Meiyao Wang, Jun Zhou, Jiachun Ge, Gangchun Xu, Yongkai Tang

**Affiliations:** 1 Key Laboratory of Freshwater Fisheries and Germplasm Resources Utilization, Freshwater Fisheries Research Center, Chinese Academy of Fishery Sciences, Ministry of Agriculture and Rural Affairs, Wuxi 214081, China; wangmy@ffrc.cn; 2 Wuxi Fisheries College, Nanjing Agricultural University, Wuxi 214081, China; 3 Freshwater Fisheries Research Institute of Jiangsu Province, Nanjing 210017, China; finedrizzle@163.com (J.Z.); gjc09@sina.com (J.G.)

**Keywords:** *Eriocheir sinensis*, cerebral ganglia, alkalinity stress, *bursicon*, signal transduction

## Abstract

(1) Background: Global climate change is intensifying, and the vigorous development and utilization of saline–alkali land is of great significance. As an important economic aquatic species in the context of saline–alkali aquaculture, it is highly significant to explore the regulatory mechanisms of *Eriocheir sinensis* under alkaline conditions. In particular, the brain (cerebral ganglion for crustaceans) serves as a vital regulatory organ in response to environmental stress; (2) Methods: In this study, a comparative transcriptome approach was employed to investigate the key regulatory genes and molecular regulatory mechanisms in the cerebral ganglion of *E. sinensis* under alkaline stress. (3) Results: The results demonstrated that the cerebral ganglion of *E. sinensis* exhibited a positive response to acute alkaline stress. Pathways associated with signal transduction and substance transportation, such as “phagosome” and “regulation of actin cytoskeleton”, along with regulatory genes involved in antioxidation, were upregulated synergistically to maintain homeostasis under alkaline stress. Furthermore, it was discovered for the first time that *bursicon* plays a positive regulatory role in the adaptation of *E. sinensis* to alkalinity. (4) Conclusions: The present study elucidates the molecular regulatory pattern of the cerebral ganglion in *E. sinensis* under acute alkaline stress as well as revealing a novel role of *bursicon* in facilitating adaptation to alkalinity in *E. sinensis*, providing valuable theoretical insights into the molecular regulatory mechanisms underlying the responses of cerebral ganglia to saline–alkali environments. These findings also offer a theoretical reference for promoting the sustainable development of the *E. sinensis* breeding industry under saline–alkali conditions.

## Simple Summary

In recent years, with the intensification of the greenhouse effect, the development and utilization of saline–alkali land distributed worldwide has gradually become a global focus. *Eriocheir sinensis*, an economically important aquatic species, has been recognized as a suitable candidate for saline–alkali aquaculture due to its strong osmotic adaptation capability in such environments. Therefore, it is crucial to explore the alkaline stress-associated regulation mechanisms of *E. sinensis*. In this study, comparative transcriptomics analysis was employed to investigate the regulatory mechanisms underlying alkaline stress in the cerebral ganglion of *E. sinensis*. Our results demonstrated a positive response of the *E. sinensis* cerebral ganglion to acute alkaline stress. Additionally, we report for the first time that *bursicon*-α and *bursicon*-β—known for their crucial role in molting development in crustaceans—exhibit significant modulatory effects in *E. sinensis* under alkaline stress conditions. Homologous cloning of *bursicon* was performed, and our findings indicate phylogenetic conservation of *bursicon* in *E. sinensis*. In summary, this study elucidates the molecular regulatory pattern of cerebral ganglia in *E. sinensis* under acute alkaline stress and reveals a novel function of *bursicon* in the adaptation of this crab to alkalinity.

## 1. Introduction

With the escalation of global warming, the salinization of land and water is becoming increasingly severe, leading to a reduction in both the area of arable land and freshwater aquaculture resources. The decrease in cultivated land area and freshwater aquaculture water resources directly leads to decreases in the grain sowing area and the volume of freshwater aquaculture water resources, leading to declines in the output and quality of food and aquatic products. In addition, the increase in saline–alkaline land and water body areas will affect cultivated land, leading to a remarkable change in the quality of water used for aquaculture and causing lasting harm to the production of food and aquatic products [1]. Therefore, actively developing and utilizing saline–alkali land is of immense significance [2,3,4]. The Chinese mitten crab (*Eriocheir sinensis*), renowned for its delicacy, nutritional richness, and remarkable stress resistance [5,6,7], serves as an economically important crustacean species. While *E. sinensis* is distributed throughout the world, it is mainly produced in Asia, especially China. In the year 2023, the output of *E. sinensis* reached 815,000 tons; its direct output value was up to 80 billion yuan [8,9]. Therefore, it is crucial to explore the influence mechanisms of saline–alkali environments on *E. sinensis*. To date, limited research has been conducted regarding the regulation mechanism of alkaline environments on *E. sinensis*, and previous studies have primarily focused on elucidating the regulatory mechanism in the gill and hepatopancreas of *E. sinensis* under alkaline conditions [10,11]. Research on the effects of alkaline stress on the hepatopancreas in *E. sinensis* showed that, in the early stage of alkaline stress, antioxidant regulation and autophagy-related genes were upregulated, thus inducing autophagy [10]. A study on the regulatory mechanism of alkaline stress on *E. sinensis* gills indicated that, under high alkaline stress, the “steroid hormone biosynthesis”, “phenylalanine, tyrosine, and tryptophan biosynthesis”, and “carbon metabolism” signaling pathways can help to resist alkaline stress through mobilizing energy reserves and inhibiting protein catabolism [11].

As a core regulatory organ, the brain plays a pivotal role in the development and anti-stress responses of organisms [12,13]. To date, limited studies have focused on the investigation of cerebral ganglia in *E. sinensis* [14,15]. These studies considered the differential regulation mechanism of the cerebral ganglion of precocious *E. sinensis* and male/female adult crabs. There have been few reports about the regulatory mechanisms of environmental stress on the cerebral ganglion of *E. sinensis*. In this study, comparative transcriptomics analysis was employed to elucidate the molecular response mechanism of the cerebral ganglion in *E. sinensis* under acute alkaline stress. This study aimed to unveil the regulation patterns associated with signal transduction, substance transport, and antioxidant response while identifying key functional regulatory genes, with the aims of revealing the anti-stress response mechanism of the cerebral ganglion of *E. sinensis* under acute alkaline stress, providing important reference information for the exploration of the response mechanism of the cerebral ganglion of *E. sinensis* under chronic alkaline stress, offering a useful reference for breeding new alkaline-tolerant varieties of *E. sinensis*, and providing valuable reference information for promoting the saline–alkali aquaculture development of *E. sinensis*.

## 2. Materials and Methods

### 2.1. Experimental Crabs, Alkaline Stress, and Sample Collection

The experimental crabs were healthy juvenile *E. sinensis* (average weight: 54.58 ± 3.13 g) with strong vitality, sound limbs, and similar specifications, obtained from the breeding base of Jiangsu Haorun Biotechnology Co., Ltd. (Taizhou, China). Three parallel subgroups were established for both the control group and the alkaline-stressed group, with each subgroup containing three male and three female individuals. Feeding was conducted twice daily (at 9 a.m. and 2 p.m.), followed by the removal of the remaining bait and feces one hour later. The feed was granular feed exclusive for *E. sinensis*, with a feed rate of 10% of body weight. The water used for the cultivation of *E. sinensis* was aerated tap water, and the daily water exchange rate was half of the total water volume, maintaining a constant water temperature of 21 ± 1 °C. Water quality parameters, including ammonia, nitrite, and dissolved oxygen, were monitored daily using water quality monitoring kits (Sampux, Beijing, China). Feeding was ceased one day prior to the initiation of the experiment. According to the study on alkaline toxicity in *E. sinensis* conducted by Yang et al. [16], sodium bicarbonate was used as the alkalinity factor in order to adjust the alkalinity to 60 mmol/L, and samples were collected at 6 h to explore the effects of acute high alkalinity on *E. sinensis*. The total alkalinity was determined via the methyl orange hydrochloride calibration method [17]. After the six-hour period of alkaline stress, one pair of male and female *E. sinensis* from each parallel subgroup in both the control and experimental groups was randomly selected and placed under anesthesia with MS-222 solution at a concentration of 40 mg/L (Kuer Bioengineering, Beijing, China) and then dissected for sampling. After removing the upper half of the carapace and the internal tissue, the cerebral ganglion was revealed, which needed to be quickly collected. The cerebral ganglia of a pair of *E. sinensis* were taken as one sample and rapidly frozen in liquid nitrogen before being stored at −80 °C for subsequent high-throughput sequencing.

### 2.2. RNA Extraction, Library Construction, and Illumina Sequencing

The total RNA was extracted using the RNAiso plus reagent (TaKaRa, Kyoto, Japan), following the manufacturer’s instructions. Subsequently, the quantification and purity of RNA were assessed using a NanoDrop 2000 spectrophotometer (Thermo Scientific, Waltham, MA, USA). Furthermore, the integrity of RNA was evaluated utilizing the Agilent 2100 Bioanalyzer (Agilent Technologies, Santa Clara, CA, USA). To enrich eukaryotic mRNA, magnetic beads with Oligo(dT) were employed, followed by fragmentation to generate cDNA libraries. Finally, sequencing was performed on the Illumina Novaseq 6000 platform (Illumina, San Diego, CA, USA).

### 2.3. Data Pre-Processing and Assembly

The Trimmomatic software (v 0.36) was employed for quality control, eliminating low-quality bases and N-bases to obtain high-quality clean reads. The Trinity software (version: 2.4.2) [18] was utilized to assemble the clean reads into transcripts, with the longest transcripts designated as unigenes.

### 2.4. Functional Annotation

Unigenes were annotated to the NR, COG/KOG, and Swissprot databases using Blastx [19] (threshold value e < 1 × 10^−5^). Based on the Swissprot annotation results, gene ontology (GO) annotation was performed according to the mapping relation between SwissProt and GO terms. Finally, the unigenes were mapped to the Kyoto Encyclopedia of Genes and Genomes (KEGG) database [20] to obtain their pathway information.

### 2.5. Quantitative and Differential Enrichment Analysis

Quantitative analysis of unigenes was performed using Bowtie 2 [21], eXpress [22], and DESeq [23]. The nbinomTest function in DESeq was employed to calculate the *p*-value and fold change for differential comparison analysis. Differentially expressed genes (DEGs) were screened based on the criteria of *p* < 0.05 and |log_2_foldChange| > 1. GO and KEGG pathway enrichment analyses of DEGs were performed using R (v 3.2.0). In transcriptomics, the top 30 GO entries and the top 10 KEGG pathways are the entries with the most significant differential expression, which can best reflect the regulatory mode of experimental factors on tissues; therefore, they were selected for analysis. The GO entries whose DEGs were more than two in three categories (Biological Process, BP; Molecular Function, MF; Cellular Component, CC) were selected as the differential GO entries. The top 30 GO entries were ranked based on the −log_10_*p*value of each entry in descending order. Similarly, the top 10 KEGG pathways were identified.

### 2.6. Validation of DEGs

Twelve DEGs were randomly selected for qRT-PCR verification. Then, Pearson correlation analysis was carried out for these DEGs to check the consistency of the qPCR results with the high-throughput sequencing results. The primer sequences are provided in the Supplementary Materials (Table S1).

### 2.7. Sequence and Phylogenetic Analysis on Key Alkaline Stress-Resistant Genes Bursicon-Alpha and Bursicon-β

*Bursicon*-α and *bursicon*-β are crucial regulatory genes for crustacean development, which are known to play significant roles in osmotic regulation [24,25]. In this study, we assessed their involvement in the alkaline tolerance of *E. sinensis*. The ORF region sequences of *bursicon*-α and *bursicon*-β from *E. sinensis* were obtained through homologous cloning (MT186753.1, MT186754.1). Multiple alignment analysis using DNA STAR7.1 was performed on *bursicon* sequences from reported species in the NCBI database, followed by motif prediction. The Mega7.0 software was applied to construct a phylogenetic tree of the reported *bursicons* using the neighbor-joining method. For the parameter settings, the “Poisson model” was selected, “rates among sites” was set as “uniform rates”, and “gaps/missing data treatment” was set as “pairwise deletion”.

### 2.8. Statistical Analysis

The statistical analysis was conducted using SPSS Statistics 26.0 and GraphPad Prism 8.0, with the results presented as the mean ± standard error of the mean (SEM). The normality of distributions was assessed using the Shapiro–Wilk test. Student’s *t*-test was employed for statistical analysis, and a significance level of *p* < 0.05 was set.

## 3. Results

### 3.1. Statistical Analysis of High-Throughput Sequencing Data

**Figure 1:**
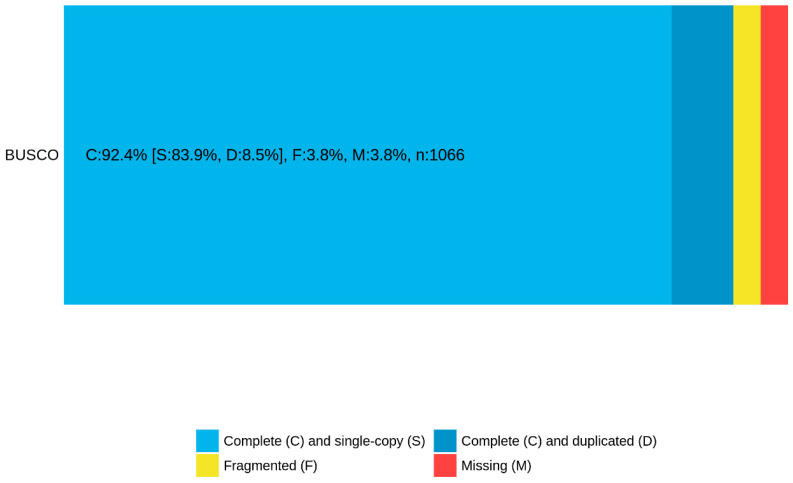
BUSCO assessment.

The Q30 values of clean reads obtained from RNA-seq, as presented in Table 1, all exceeded 90%. Furthermore, the BUSCO analysis results (depicted in Figure 1) revealed a remarkable proportion of complete sequences (92.4%) with minimal duplication (8.5%). Collectively, these findings affirm the reliability of our high-throughput sequencing results.

### 3.2. Top 30 GO Enrichment Analysis of Cerebral Ganglia in E. sinensis Under Acute Alkaline Stress

**Figure 2:**
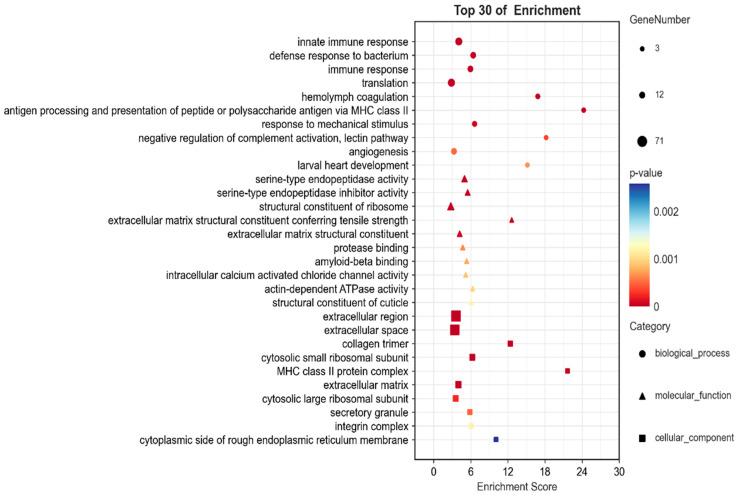
Differential expression enrichment analysis on the top 30 GO terms.

As depicted in Figure 2, the top 10 BP terms primarily encompassed immune regulation, stress response, information processing, and development. The top 10 MF terms predominantly involved stress adaptation, energy metabolism, protein synthesis, signal transduction, and developmental regulation. The top 10 CC terms mainly comprised extracellular region, ribosome, endoplasmic reticulum structure, collagen trimer domain, secretory vesicle, integrin, and so on, which were associated with protein synthesis and processing as well as substance transport and information presentation. The overall findings indicated that the top 30 GO entries were primarily involved in the anti-stress response, along with regulation of substance transport and development.

### 3.3. Differential Enrichment Analysis of the Top 10 KEGG Pathways

**Figure 3:**
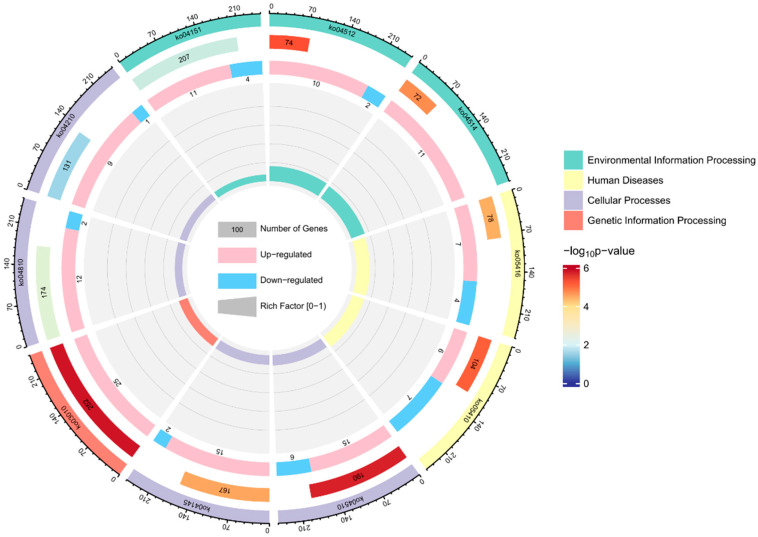
Top 10 KEGG analysis. The results were shown in four circles from outside to inside. First circle: Classifications of top 20 pathways. Coordinatometer indicated numbers of DMs. Second circle: Number of DMs and *p*-value of pathways. Longer bars indicate more DMs. Third circle: the proportion of upregulated and downregulated DMs; red represents upregulation and blue represents downregulation. Fourth circle: Rich factor for the top 20 pathways. Each gray auxiliary line represents an increment of 0.2.

The top 10 KEGG pathways in response to acute alkaline stress, as depicted in Figure 3, primarily involved the regulation of substance transport, cell metabolism, and signal transduction. Furthermore, the majority of DEGs exhibited upregulation in the various pathways.

### 3.4. Key DEGs

A comprehensive analysis of the top 30 GO terms and the top 10 KEGG pathways revealed that key DEGs were primarily associated with regulation of substance transport, signal transduction, and anti-stress and antioxidant responses. The corresponding DEGs for each functional category are presented in Table 2.

### 3.5. qRT-PCR Validations

**Figure 4:**
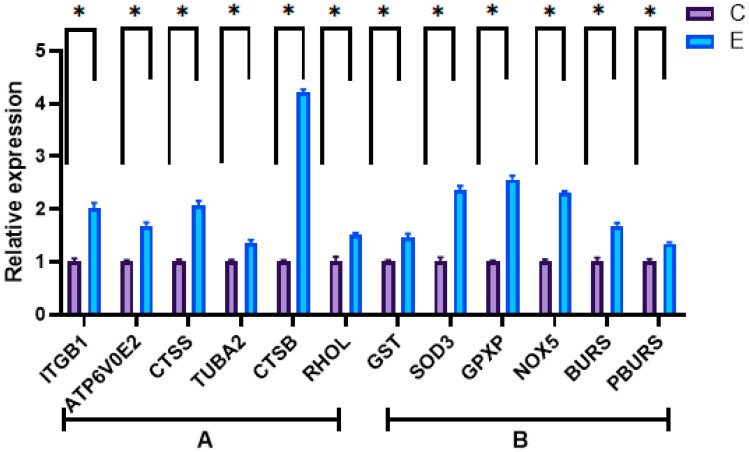
qPCR validation. Letters “A” and “B” represent two functional gene clusters of DEGs. “A” represents “substance transport and signal transduction” and “B” represents “anti-stress and antioxidant response”. “C” represents the control group and “E” represents the experimental group. “*” indicated significant difference, *p* < 0.05.

**Figure 5:**
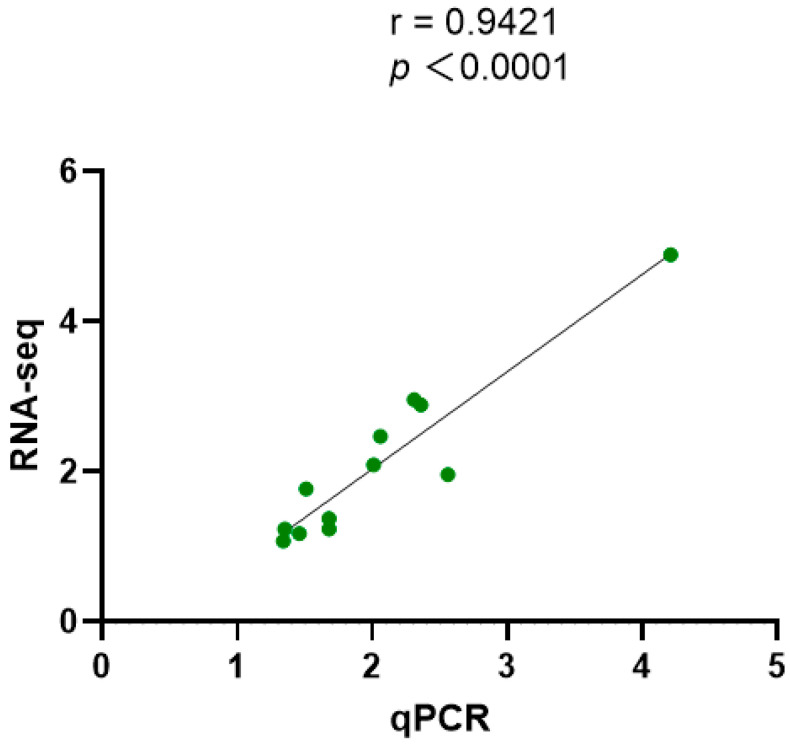
Pearson correlation analysis. The dots represented the detected DEGs.

Six DEGs were randomly selected from each of the two aforementioned functional gene groups. As shown in Figure 4, there was a significant disparity in expression between the control and experimental groups. In addition, Pearson correlation analysis demonstrated that there was a significant correlation between RNA-seq and qPCR data (Figure 5), collectively affirming the accuracy of the RNA-seq data.

### 3.6. Sequencing and Phylogenetic Analysis of Bursicon-α and Bursicon-β

**Figure 6:**
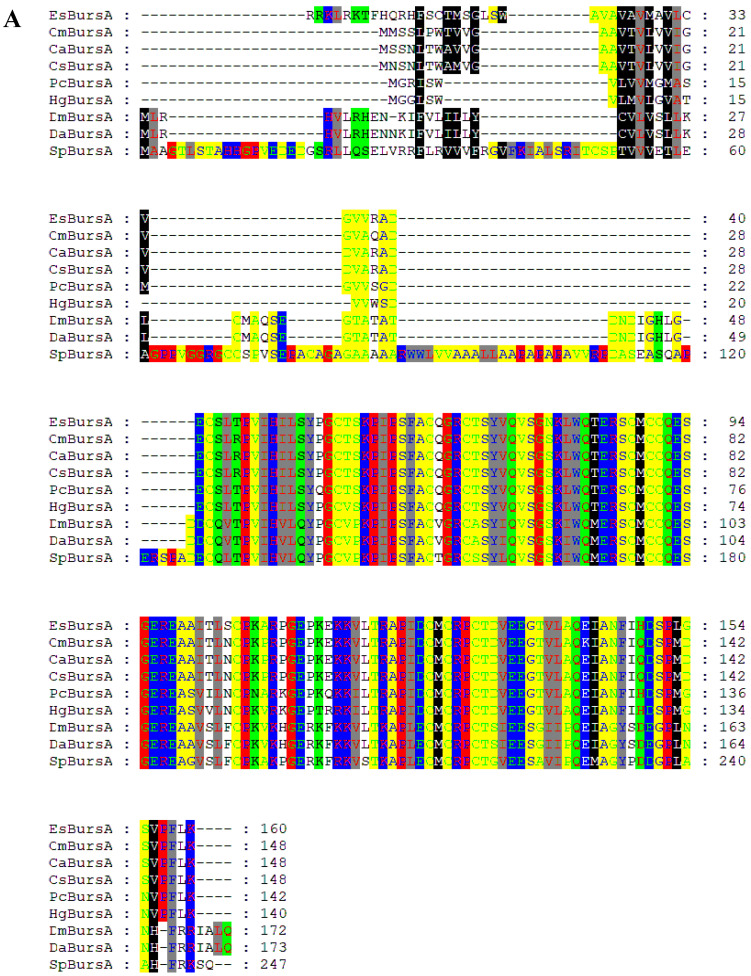

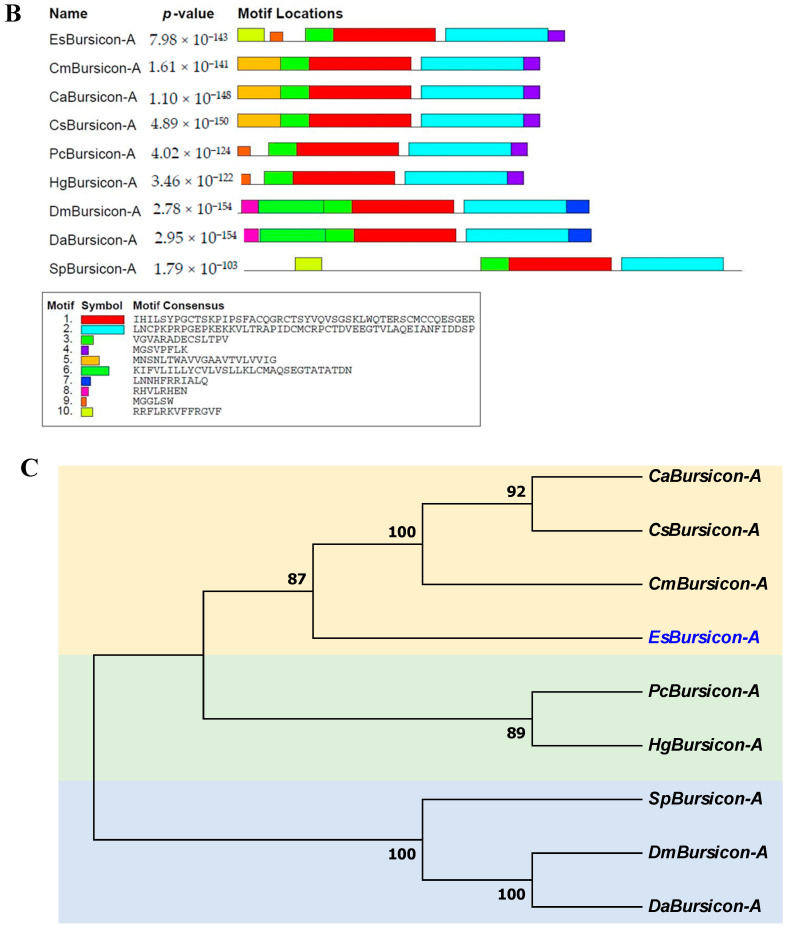
Sequence and phylogenetic analysis of *bursicon*-α in *E. sinensis*. (**A**) The multiple alignment of *bursicon*-α sequences. The conserved amino acid residues were highlighted in colored shadow. Species names were abbreviated as follows: EsBursA, *Eriocheir sinensis bursicon*-α; Cm, *Carcinus maenas*; Ca, *Callinectes arcuatus*; Cs, *Callinectes sapidus*; Pc, *Procambarus clarkii*; Hg, *Homarus gammarus*; Dm, *Drosophila mojavensis*; Da, *Drosophila arizonae*; Sp, *Schistocerca piceifrons*. (**B**) Analysis of motifs of reported *bursicon*-α sequences. Different motifs were identified in distinct colors. (**C**) phylogenetic tree of *bursicon*-α. The phylogenetic tree was constructed with the NJ (neighbor-joining) method. The values at the nodes indicate the bootstrap percent when bootstrap replications reach 1000.

**Figure 7:**
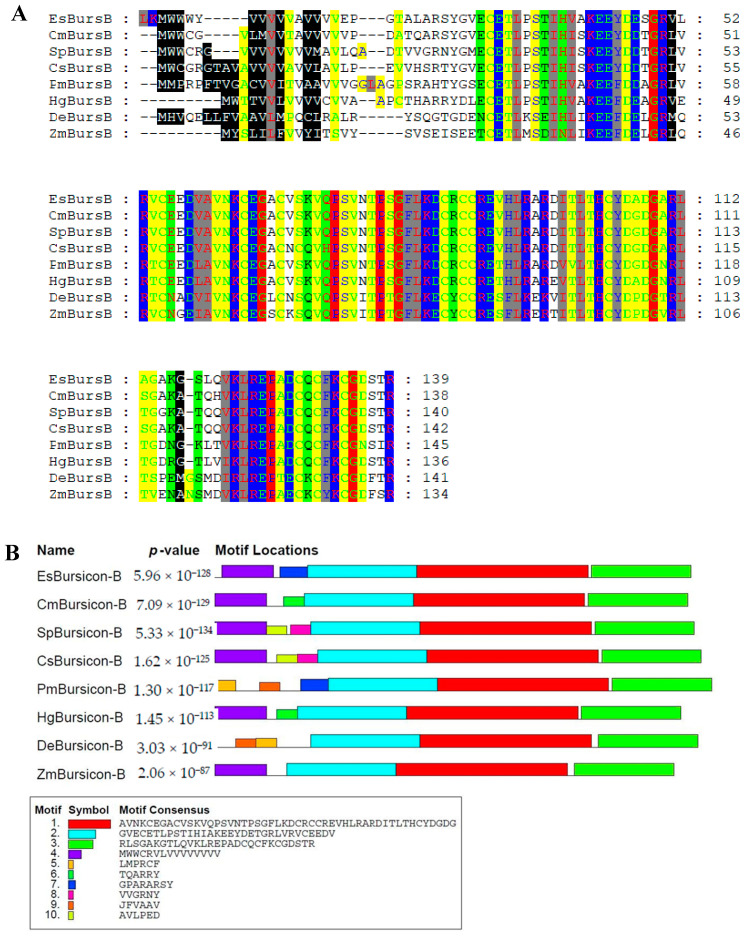

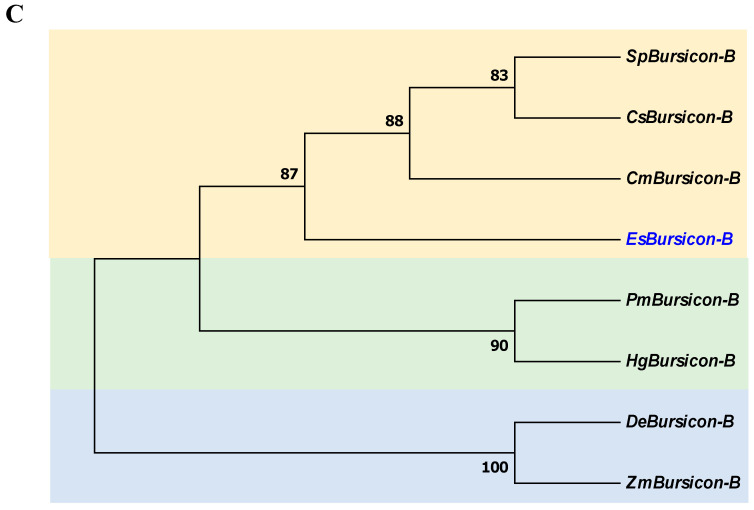
Sequence and phylogenetic analysis of *bursicon*-β in *E. sinensis*. (**A**) The multiple alignment of *bursicon*-β sequences. The conserved amino acid residues were highlighted in colored shadow. Species names were abbreviated as follows: EsBursA, *Eriocheir sinensis bursicon*-α; Cm, Carcinus maenas; Ca, *Callinectes arcuatus*; Cs, *Callinectes sapidus*; Pc, *Procambarus clarkii*; Hg, *Homarus gammarus*; Dm, *Drosophila mojavensis*; Da, *Drosophila arizonae*; Sp, *Schistocerca piceifrons*. (**B**) Analysis of motifs of reported *bursicon*-β sequences. Different motifs were represented in distinct colors. (**C**) Phylogenetic tree of *bursicon*-β. The phylogenetic tree was constructed with the NJ (neighbor-joining) method. The values at the nodes indicate the bootstrap percent when bootstrap replications reach 1000.

The results of the multiple alignment of *bursicon*-α and *bursicon*-β are presented in Figure 6 and Figure 7. As shown in Figure 6A and Figure 7A, the sequences of *bursicon*-α and *bursicon*-β in *E. sinensis* exhibited a high degree of conservation. Furthermore, motif analysis revealed conserved motifs between *bursicon* in *E. sinensis* and those reported in other species (Figure 6B and Figure 7B). Additionally, the phylogenetic tree demonstrated that the *bursicon* of *E. sinensis* clustered with that of other crab species before grouping with shrimps. Crustaceans, including shrimp and crab species, formed one branch, while insects constituted another branch.

## 4. Discussion

### 4.1. Regulation of Cellular Substance Transport and Signal Transduction in the Cerebral Ganglia of E. sinensis Under Acute Alkaline Stress

**Figure 8:**
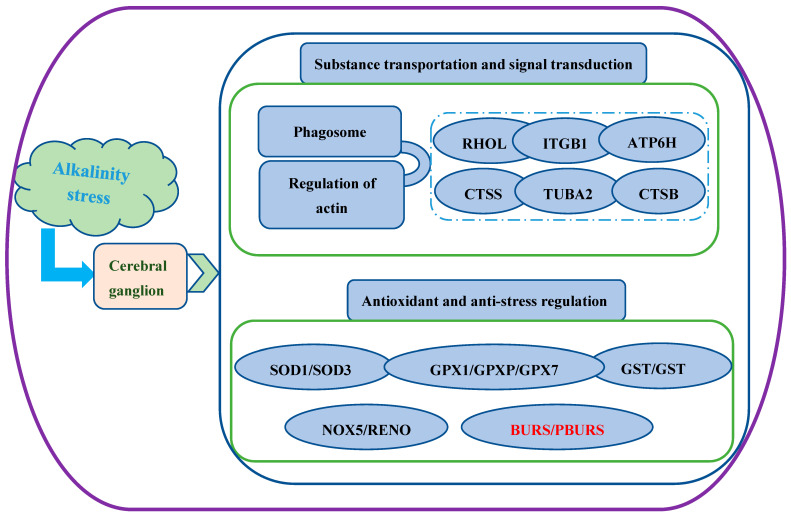
Schematic diagram of the regulatory pattern on the cerebral ganglion of *E. sinensis* under acute alkalinity stress.

Following exposure to alkaline stress, the substance transport pathway (“phagosome”) and signal transduction pathway (“regulation of actin cytoskeleton”) in the cerebral ganglion of *E. sinensis* were significantly upregulated (Figure 8). Phagocytosis serves as a crucial mechanism for transmembrane substance transport, tissue remodeling, and resistance against harmful factors [26]. The actin cytoskeleton, composed of actin and related regulatory proteins, plays diverse physiological roles such as maintaining cell morphology, facilitating cell movement, and enabling environmental adaptation. Conversely, its dysfunction can lead to diseases such as nervous system disorders and cardiovascular disease [27]. Studies on alkaline exposure effects on the gills of oriental river prawns (*Macrobrachium nipponense*) have demonstrated that the phagosome pathway positively regulated adaptation to alkalinity [28]. Comparative transcriptome studies on saline–alkali stress in naked carp (*Gymnocypris przewalskii*) have indicated that actin cytoskeleton regulation plays a vital role in maintaining homeostasis under saline–alkali stress conditions [29]. Lysosomes can decompose various endogenous and exogenous substances, such as lipids, proteins, and so on, and play important roles in signal transduction, plasma membrane repair, and maintenance of cellular homeostasis [30]. Cathepsins are the most abundant lysosomal proteases, which play important roles in intracellular protein degradation and energy metabolism. Cathepsin B is a lysosomal cysteine protease; as an important member of the cathepsins family, it plays an essential role in the lysosomal protein degradation pathway, maintaining an accurate balance between protein synthesis and degradation. It is necessary for lysosomal function and the even maintenance of homeostasis [31]. An alkaline stress study conducted in Nile tilapia revealed a downregulation of cathepsin B due to damage to the fish caused by exposure to alkalinity [32]. In this study, the upregulation of cathepsin B contributed to the regulation of cerebral ganglion cell homeostasis.

Ras-like GTP-binding protein (RHOL) is a member of a large family of small GTP-binding proteins, which have GTP hydrolase activity and are distributed on the cytoplasmic side of the plasma membrane. RHOL can activate many important downstream signaling pathways, such as the MAPK pathway, and plays important regulatory roles in cell growth, differentiation, cytoskeleton, protein transport and secretion, and so on. Therefore, RHOL has an important regulatory role in the responses of organisms to environmental stresses, such as alkalinity [33]. Studies on the regulatory mechanism under exposure to alkalinity in *Macrobrachium nipponense* have indicated that alkaline stress upregulated RHOL in the gill, which played a positive regulatory role in the anti-alkalinity response [28]. In this study, a similar expression pattern of RHOL was observed in the cerebral ganglion of *E. sinensis*, facilitating its adaptive regulation under alkalinity stress. The V-type proton ATPase subunit (ATP6V0E2) can reduce the damage caused by environmental stress by regulating its conformation and then changing the structure, state, and quantity of enzymes [34]. A previous report has highlighted the significant regulatory role of a ATP6V0E2 as an ion transport protein in *Exopalaemon carinicauda* during acute alkalinity stress [34]. Similarly, upregulation of RHOL was found to positively regulate the adaptation of *E. sinensis* to alkalinity in this study. Furthermore, our findings revealed the upregulation of pathways such as “phagosome” and “regulation of actin cytoskeleton”, along with corresponding DEGs, suggesting an active regulation of cytoskeleton dynamics, substance transport homeostasis, and signal transduction in the cerebral ganglion of *E. sinensis* during the response to alkaline stress.

### 4.2. Antioxidant and Anti-Stress Responses Under Alkaline Stress

In this study, a significant upregulation of regulatory genes associated with the antioxidant system, including glutathione S-transferase (GST) and glutathione peroxidase 1 (GPX), was observed under acute alkaline stress. GST is an important detoxification enzyme, whose main function is to catalyze the combination of some endogenous or exogenous electrophilic groups of harmful substances with the sulfhydryl group of reduced glutathione. It can catalyze the initial step of the glutathione binding reaction to form more soluble and non-toxic derivatives, which are easier to excrete in vitro or be decomposed by phase III metabolites. GST plays a pivotal role in regulating resistance to environmental stressors and homeostasis maintenance [35,36]. Transcriptome analysis investigating the adaptation to alkalinity in *Leuciscus waleckii* identified GST as an important differentially expressed gene involved in alkalinity adaptation [37]. A physiological study on olive flounder (*Paralichthys olivaceus*) has demonstrated that the GST expression level is sensitive to environmental pH variations [38].

The enzyme glutathione peroxidase (GPX) is capable of converting toxic peroxides into non-toxic hydroxyl compounds, thereby safeguarding the integrity and functionality of the cell membrane against oxidative interference and damage, maintaining organismal homeostasis [39]. Previous research on *E. sinensis* has revealed that exposure to alkaline stress can induce upregulation of the antioxidant enzyme GPX in the hepatopancreas [10]. Similarly, in this study, as a key regulator of antioxidation, the expression of GPX in the cerebral ganglion of *E. sinensis* was significantly upregulated after acute alkaline stress. In addition, an investigation on the regulatory mechanisms in the gill of *Macrobrachium nipponense* under alkaline stress has highlighted the crucial role that GPX plays in maintaining homeostasis [28]. In this study, we observed the upregulation of antioxidant genes, including GST and GPX, in the cerebral ganglia of *E. sinensis* under acute alkaline stress, indicating their active involvement in facilitating adaptation to alkalinity.

In addition, we also observed upregulation of key regulatory genes involved in development and anti-stress responses, including the *bursicon*-alpha subunit (BURS) and *bursicon*-beta subunit (PBURS). *Bursicon* is a conserved heterodimeric neuropeptide hormone composed of α and β subunits [40,41], which directly regulates carapace development. Previous studies on crustaceans such as shore crab (*Carcinus maenas*) and blue crab (*Callinectes sapidus*) have demonstrated the crucial role of *bursicon* in molting development [24,42]. Additionally, research on *Macrobrachium rosenbergii* has revealed that *bursicon* not only plays a significant role in molting development but also contributes to the response against environmental factors such as salinity [25]. Our findings indicated that the substantial upregulation of BURS and PBURS in the cerebral ganglion of *E. sinensis* positively influences its adaptation to alkalinity.

## 5. Conclusions

The cerebral ganglion of *E. sinensis* exhibited an upregulation of genes involved in the cytoskeletal components, substance transport, and signal transduction regulatory pathways, such as phagosome and regulation of actin cytoskeleton, under acute alkaline stress. Furthermore, it was discovered for the first time that *bursicon*-α and *bursicon*-β, which play crucial regulatory roles in molting development and anti-stress responses, exhibit positive regulatory roles in the adaptation of *E. sinensis* to alkaline conditions. This study provides new insights for the establishment of target genes enabling the breeding of saline–alkaline-tolerant *E. sinensis* in the future, as well as a theoretical reference for the development of *E. sinensis* aquaculture under saline–alkaline environments.

## Figures and Tables

**Table 1:** Transcriptome sequencing quality information of the samples.

Sample	RawReads (M)	CleanReads (M)	ValidBases (%)	Q30 (%)	GC (%)
C1	46.69	45.43	93.25%	93.20%	49.93
C2	50.03	48.69	90.34%	94.27%	49.91%
C3	47.58	46.11	92.18%	94.03%	48.97
E1	47.44	46.17	90.38%	95.12%	49.91
E2	47.33	45.95	91.82%	93.06%	49.20
E3	48.90	47.23	93.02%	94.17%	49.61

Note: C1–C3 represented three samples in the control group; E1–E3 represented the samples in the experimental group. Q30 refers to the proportion of bases with a base error rate of 0.1%.

**Table 2:** Key DEGs in the cerebral ganglion of *E. sinensis*.

Category	Gene Name	Gene Definition	log_2_Foldchange	*p*-Value
Substance transport and signal transduction	RHOL	Ras-like GTP-binding protein	1.7742	8.62 × 10^−7^
	ITGB1	Integrin beta 1	2.0946	2.90 × 10^−6^
	ATP6V0E2	V-type proton ATPase subunit e 2	1.375	1.53 × 10^−6^
	CTSS	Cathepsin S	2.4724	0.000771
	TUBA2	Tubulin alpha-2	1.2379	1.25 × 10^−5^
	CTSB	Cathepsin B	4.8926	0.000704
	TUBA1B	Tubulin alpha-1B	1.0039	3.84 × 10^−5^
Anti-stress and antioxidant response	SOD1	Superoxide dismutase [Cu-Zn]	1.614355	5.29 × 10^−6^
	GST	Glutathione S-transferase	1.178236	0.033669
	SOD3	Extracellular copper/zinc superoxide dismutase 3	2.891303	0.0001
	GPX1	Glutathione peroxidase 1	2.518527	0.00328
	GPXP	Glutathione peroxidase 3	1.963184	0.000246
	GPX7	Glutathione peroxidase 7	1.236985	0.008292
	GSTM2	Glutathione S-transferase	1.469546	0.007026
	NOX5	NADPH oxidase 5	2.961806	7.62 × 10^−9^
	RENOX	NADPH oxidase 4	1.543761	0.000511
	BURS	*Bursicon*-alpha subunit	1.785945	0.036662
	PBURS	*Bursicon*-beta subunit	1.528678	0.021648
	TIMP3	Metalloproteinase inhibitor 3	−1.07	0.03

## Data Availability

Raw data have been submitted to the NCBI public database (PRJNA1178569). All other data are contained within the main manuscript.

## Supplementary Materials

The following supporting information can be downloaded at: https://www.mdpi.com/article/10.3390/biology14010084/s1, Table S1. The genes and primers used for Real-time RT-PCR validation.
