# Supplementary material for: Molecular Mechanisms Underlying Substance Transport, Signal Transduction, and Anti-Stress Regulation, as Well as Anti-Alkaline Regulation via Bursicon in the Cerebral Ganglion of Chinese Mitten Crab Eriocheir sinensis Under Alkaline Stress
Source: Biology (Basel). 2025 Jan 16;14(1):84. doi: 10.3390/biology14010084 (PMC11762361; doi:10.3390/biology14010084)
Supplement: Supplementary file 1 [file biology-14-00084-s001.zip › biology-3357947-supplementary.pdf]

Supplementary file

Table S1. The genes and primers used for Real-time RT-PCR validation

Number	Gene name	Gene definition	Sequence(5’→3’)
1	ITGB1	Integrin beta 1	F: ACCCCATCTACCTGCAGAAC<br>R: TGCTTATGTTCTGGGCCTCA
2	ATP6V0E2	V-type proton ATPase subunit e 2	F: GTGGCCGTCATGACCTCC<br>R: CGGGTTCATCTGATGCATGT
3	CTSS	Cathepsin S	F: TCAAGAATGCAGAGGGCAAG<br>R: AGGTCCCTTATTGGCCACAG
4	TUBA2	Tubulin alpha-2	F: CCATCCTGACAACACACACG<br>R: TCAGGGATGCTGTGATGGAG
5	CTSB	Cathepsin B	F: CTCAAGGTGTCGCAGGCC<br>R: CAAGCCCACCCGGTTCAG
6	RHOL	Ras-like GTP-binding protein	F: ACGGCGTGGAGTACAACTAT<br>R: GCCACACTCCTTCCTGATCT
7	GST	Glutathione S-transferase	F: TGTGTTCCCAGCATTGTTCC<br>R: ACAGTGGTAAGGAGGGTGTG
8	SOD3	Extracellular copper/zinc superoxide dismutase 3	F: TCGGAATGAAGCTGCTCCTC<br>R: AGCCATGAAGACCCTGAGAC
9	GPXP	Glutathione peroxidase 3	F: CCAAGGCCTGAATGTGCTAC<br>R: CGTTCTTTCCGTTGACCTCG
10	NOX5	NADPH oxidase 5	F: TTGGAGATTGAGCAGGCAGA<br>R: GCGTTGGTTCTGGTCTTGAG
11	BURS	Bursicon alpha subunit	F: GACACTTCAGCTGCACGATG<br>R: AGACACCTGGACGTACGAAG
12	PBURS	Bursicon beta subunit	F: CTCGGTAAACACGCCTTCTG<br>R: GAGTCGCCACATTTGAAGCA
13	β-actin	Beta-actin	F: GCAACACGCAGCTCGTTGTAG<br>R: CAGGCATCAGGGTGTGATGG
